# Contrast-ultrasound dispersion imaging for renal cell carcinoma diagnostics

**DOI:** 10.1186/s13089-025-00423-7

**Published:** 2025-04-16

**Authors:** Peiran Chen, Simona Turco, Zhaohan Liu, Christiaan Widdershoven, Jorg Oddens, Hessel Wijkstra, Massimo Mischi, Patricia Zondervan

**Affiliations:** 1 Department of Electrical Engineering, Eindhoven University of Technology, De Groene Loper 19, Eindhoven, 5612 AP the Netherlands; 2 Department of Urology, Amsterdam University Medical Centers, De Boelelaan 1117, Amsterdam, 1081 HV the Netherlands

**Keywords:** Urology, Renal cell carcinoma diagnostics, Angiogenesis, Dynamic contrast-enhanced ultrasound, Contrast-ultrasound dispersion imaging

## Abstract

Cost-effective screening methods for Renal Cell Carcinoma (RCC) are still lacking. Angiogenesis is a recognized hallmark of cancer growth, leading to distinguishable perfusion patterns in tumors from those in normal tissue. This establishes the basis for diagnostic imaging solutions by dynamic contrast-enhanced ultrasound (DCE-US). In the past years, we have developed contrast-ultrasound dispersion imaging (CUDI) techniques to quantify prostate DCE-US acquisitions, obtaining promising results for prostate cancer localization. In this pilot study, we investigated for the first time its feasibility for RCC localization. DCE-US acquisitions of the kidney in 5 patients were used to perform CUDI analysis. With the obtained CUDI parameters and the delineated tumor and parenchyma regions, we performed pixel-based classification, from which the highest area under the receiver-operating-characteristic curve (AUC) = 0.96 was obtained for an individual patient, and an average AUC = 0.68 was obtained for the full patient dataset, showing the potential of CUDI for solid RCC localization. Further validation in a larger dataset and evaluation of the compatibility of point-of-care diagnosis are required.

## Introduction

With over 430,000 new cases and around 180,000 new deaths in 2020, kidney cancer accounts for 2.2% and 1.8% of all oncological diagnoses and deaths worldwide, respectively [1]. About nine out of ten cancers in the kidney are renal cell carcinomas (RCCs) [2]. RCC comprises a broad spectrum of histopathological entities, of which the three main RCC subtypes are: clear-cell RCC (ccRCC), papillary RCC (pRCC-type I and II), and chromophobe RCC (chRCC) [3]. Typically, RCC appears symptom-free at its early stage until progression [4]. Patients with RCCs can be cured at an early stage, while patients with stage four disease have a 12% five-year survival rate only [5]. Nowadays, over 60% of RCCs are diagnosed incidentally with computed tomography (CT) imaging performed for other medical purposes [3, 4]. Therefore, an efficient diagnostic method compatible with point-of-care diagnosis is in demand for RCC detection and screening, especially at an early stage.

Tumor-driven angiogenesis is a recognized indicator of cancer growth, which is characterized by increased microvascular density, higher vessel tortuosity, and smaller and irregular vessel diameter [6–8]. Due to the complex microvascular architecture, blood flow patterns in the malignant regions become distinguishable from those in the benign tissue regions, establishing the basis for tumor diagnostics by the assessment of blood perfusion [9–11]. According to the European Association of Urology guidelines on RCC, contrast-enhanced computed tomography (CE-CT) is strongly recommended for RCC diagnostics [3]. However, CE-CT imaging is expensive and utilizes ionizing radiations. Being cost effective and radiation-free, dynamic contrast-enhanced ultrasound (DCE-US) imaging represents a promising alternative. DCE-US provides real-time analysis of the tumor (micro)vasculature and enhancement characteristics by imaging the intravascular passage of ultrasound contrast agents (UCAs) following their intravenous injection.

In clinical routine, the diagnosis of RCCs using DCE-US imaging is mostly based on qualitative evaluation of the enhancement patterns in the kidney during different vascular phases [12–14]. However, qualitative assessment suffers from inter-observer variability [15]. More recently, quantitative analysis of DCE-US acquisitions has been proposed by extraction of perfusion parameters from the time-intensity curves (TICs) reflecting the temporal evolution of the UCA concentration [16, 17]. However, this assessment can be influenced by ultrasound attenuation, scanner settings, and the complex, inconsistent correlation between angiogenesis and perfusion [18, 19].

To overcome these limitations and achieve the goal of an efficient diagnostic method by DCE-US, we have proposed and developed contrast-ultrasound dispersion imaging (CUDI) to quantitatively analyze DCE-US acquisitions by modeling the kinetics of UCAs as a convective-dispersion process. By CUDI, we have obtained promising results for prostate cancer localization in the past years [19–24]; however, investigation of the feasibility of CUDI for kidney cancer diagnosis is still lacking. Here, we translated CUDI for the first time for the quantitative analysis of DCE-US acquisitions in the kidney by optimizing DCE-US acquisitions, performing CUDI analysis, and assessing the diagnostic performance in patients, aiming at investigating the feasibility of CUDI for primary RCC diagnostics.

## Materials and methods

### Data acquisition

The data acquisition was performed on nine patients at the Amsterdam University Medical Center (UMC, location AMC) under approval granted by the local ethics committee. In this pilot study, we aimed at optimizing kidney DCE-US scan settings and investigating the feasibility of CUDI for kidney DCE-US acquisitions analysis; therefore, we focused more on the technical side rather than building a standard clinical study. For this purpose, the inclusion criteria were patients who were diagnosed with kidney cancer by CT scan and referred for surgery. All DCE-US acquisitions were performed by a Philips iU22 ultrasound scanner (Philips Healthcare, Bothell, WA) equipped with a C5-2 convex transducer, operating in power modulation at 3.5 MHz with a mechanical index of 0.19. The scan duration was 120 s following an intravenous injection of a bolus of 2.4-mL SonoVue^®^ (Bracco, Milan, Italy) UCA and a subsequent flush of 10 mL saline. During the scanning, the patient was under anesthesia for a planned radical or partial nephrectomy and a short period of timed apnea to mitigate the impact of respiratory motion. The first four acquisitions were used to optimize the ultrasound machine settings and the remaining five acquisitions were used for analysis.

### Data pre-processing

Prior to performing CUDI analysis, ultrasound data linearization was implemented to recover a linear relation between the acoustic intensity and the UCA concentration from the log-compressed DCE-US data [20]. Subsequently, the spatial resolution of the ultrasound image was first measured by computing a kernel-based autocovariance over the whole image, as described in [22, 25]. After that, speckle size regularization was performed to avoid the influence of anisotropic and depth-dependent speckle on the TIC analysis [22, 25], obtaining a regularized resolution of 0.85 mm in both the axial and lateral directions. Moreover, motion resulting from free-hand scanning was compensated using the strategy described in [26]. After that, singular value decomposition (SVD) was employed to spatiotemporally remove residual tissue clutter and noise signals while preserving UCA movement and signal intensity variations [27]. The cut-off threshold for rejecting singular values was determined on the basis of the mean frequency estimated from the power spectral density of the temporal singular vectors [28]. And for each type of analysis, SVD filtering was the same for all the acquisitions.

### TIC fitting analysis

The pre-processed data was analyzed by two different CUDI techniques. One approach comprises fitting the modified local density random walk (mLDRW) model to the TICs measured at each pixel, from which the dispersion-related parameter *κ* can be extracted as described in [20]. The mLDRW, which is an analytical solution to the convective-dispersion process, is derived as

$$\:C\left(t\right)=AUC\sqrt{\frac{\kappa\:}{2\pi\:\left(t-{t}_{0}\right)}}\text{exp}\left(-\frac{\kappa\:{\left(t-{t}_{0}-\mu\:\right)}^{2}}{2\left(t-{t}_{0}\right)}\right),$$

where *C(t)* is the measured TIC, $\:AUC$ is the area under the TIC, $\:{t}_{0}$ is the theoretical injection time of the contrast agent, and $\:\mu\:$ is its mean transit time. For the fitting, the SVD cut-off frequency was set to 0.2 Hz. In addition, typical perfusion parameters such as the appearance time (AT, the time when the intensity reaches 5% of the peak) and full-width at half-maximum (FWHM, the time duration when the intensity is above 50% of the peak) were also extracted from the fitted curves. Only pixels with sufficient fitting quality (determination coefficient $\:{R}^{2}$ > 0.7) were considered for further analysis.

### Spatiotemporal similarity analysis

Cancer-associated changes in the microvascular network can lead to tumor perfusion patterns and UCA dispersion kinetics that are distinguishable from those in normal tissue. By modelling the spatiotemporal evolution of the UCA concentration as a convective-dispersion process, the changes in perfusion and dispersion lead to different TIC shapes. Tumor growth is associated with increased blood perfusion and decreased dispersion, which is reflected into a less skewed TIC shape and reduced variation of the TIC shape over the tumor region; therefore resulting in higher similarity between neighboring TICs [19, 22]. As described in [19], spatiotemporal similarity analysis was performed by each TIC with neighboring TICs extracted from a ring-shaped kernel. The inner and outer radius of the kernel were set at 1 and 2.5 mm, respectively, accounting for the system resolution and the scale at which angiogenesis occurs. The kernel should be larger than the system resolution but smaller than the scale at which angiogenesis occurs [6]. This enabled the estimation of linear similarity measures including the spectral coherence (*ρ*) [19, 22] and the temporal correlation (*r*) [21], as well as nonlinear similarity measures such as mutual information (*I*) [23]. For spatiotemporal similarity analysis, the SVD cut-off frequency was set to 0.5 Hz, and a dedicated time window of 35 s was applied to each TIC. The role of the time window was to focus on the most relevant part of the TIC, which contains the first UCA passage [21–23].

### Method validation

**Fig. 1:**
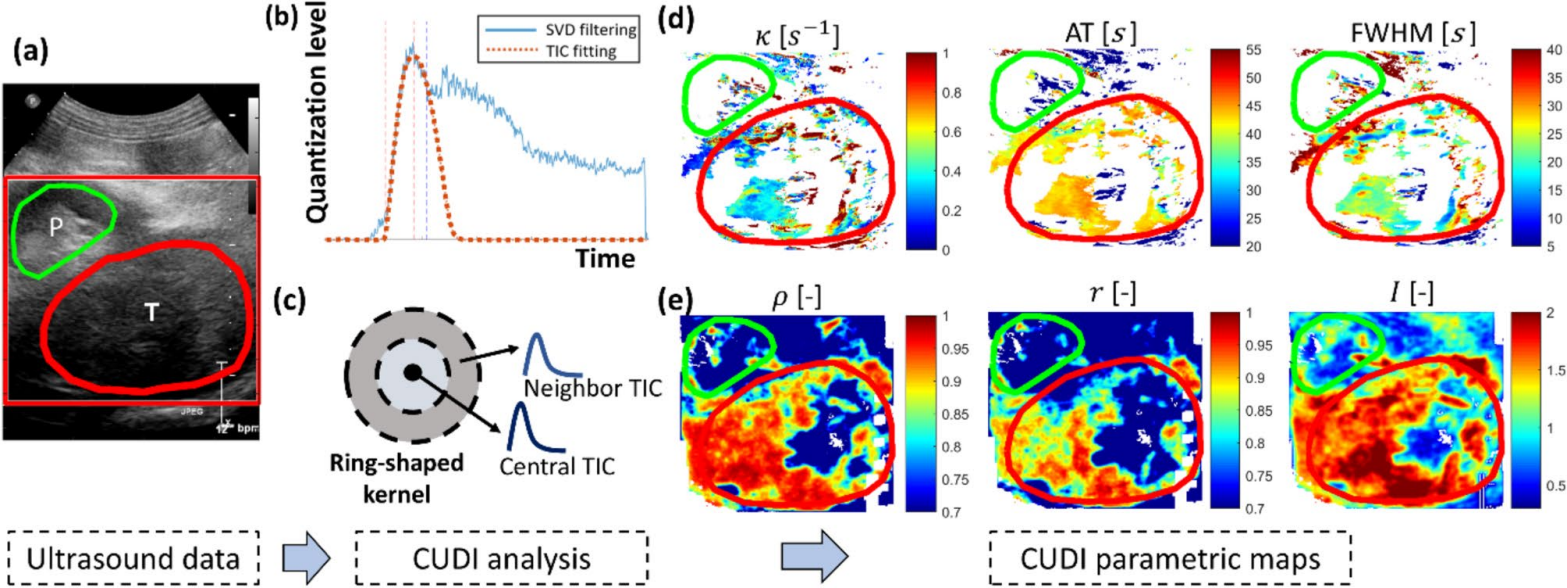
CUDI analysis of one patient dataset (case 1, ccRCC subtype). (**a**) is the B-mode image of the kidney. The tumor (T) and parenchyma (P) regions are indicated by red and green contours, respectively. (**b**) and (**c**) show the CUDI analysis comprising the TIC fitting analysis and the spatiotemporal similarity analysis. (**d**) shows the parametric maps of the TIC fitting results: *κ*, AT and FWHM. Pixels with low fitting quality ($\:{R}^{2}$ <0.7) are shown in white; (**e**) shows the parametric maps of the spatiotemporal similarity analysis results: *ρ*, *r* and *I*. Pixels with low enhancement (less than 10 gray levels) are regarded as invalid pixels and shown in white

Five DCE-US acquisitions were used to investigate the feasibility of CUDI for solid RCC diagnostics. Four cases were ccRCC histological subtype and one was pRCC. For each acquisition, tumor and parenchyma regions were delineated by two urologists in consensus, based on the corresponding ultrasound B-mode images and CT scans (Fig. 1). The CUDI parametric maps of the delineated tumor and parenchyma regions were compared. Pixel-based classification was then performed by the obtained CUDI parameters in each individual acquisition as well as in the combined dataset of the five acquisitions. Considering the different sizes of delineated tumor and parenchyma regions, the same number of pixel samples from both the delineated tumor and parenchyma regions in each patient data were randomly selected for the pixel-based classification, avoiding imbalanced classification. The pixels in the tumor and parenchyma regions were given the label as positive or negative, and the obtained CUDI parameters were used as scores. Based on this, for each CUDI parameter a receiver-operating-characteristic curve could be derived, and the area under the receiver-operating-characteristic curve (AUC) was then computed to assess the classification performance for each parameter. A statistical analysis was performed to assess the significance of the performance difference (*p*-value) between the TIC fitting and spatiotemporal similarity analysis using the single-tailed Wilcoxon signed-rank test with the AUC values corresponding to the two types of analysis as the two groups of input. An overview of the five DEC-US acquisitions is displayed in Table 1.

**Table 1:** Overview of five DCE-US acquisitions

	Case 1	Case 2	Case 3	Case 4	Case 5
Tumor histological types	ccRCC	ccRCC	ccRCC	ccRCC	pRCC
Delineated lesion diameter	6 cm	5 cm	4 cm	3 cm	3 cm
Delineated parenchyma diameter	2 cm	approx.2 cm	3 cm	4 cm	3 cm

## Results

**Fig. 2:**
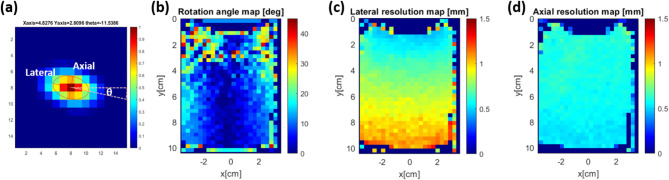
(**a**) Shows a local autocovariance result. An ellipse contour was fitted to the full-width at half-maximum of the local autocovariance result, from which the long axis, short axis, and rotation angle can then be extracted. By moving the kernel over the whole image, the distribution of the long axis, short axis, and rotation angle can be obtained, describing the variation of lateral and axial resolutions as shown in (**b**-**d**)

Figure 2 shows the measurement results of the axial and lateral resolution of the ultrasound image.

Figure 1 shows the CUDI analysis results for one ccRCC patient. The parametric maps based on the TIC fitting analysis and the spatiotemporal similarity analysis demonstrate the difference between the tumor and parenchyma regions. The TIC fitting analysis revealed distinct hemodynamic characteristics in the tumor region, characterized by higher *κ* and AT values, along with lower FWHM values. The increased *κ* and reduced FWHM values indicate rapid wash-in and wash-out kinetics of UCA following its arrival. These findings were further supported by the spatiotemporal similarity analysis, which demonstrated higher *ρ*, *r*, and *I* values, reflecting enhanced perfusion efficiency and reduced dispersion within the tumor vasculature. This hemodynamic pattern, marked by rapid contrast kinetics, is potentially indicative of the tumor’s perfusion characteristics.

Qualitatively, the parametric maps revealed that the spatiotemporal similarity analysis more clearly distinguishes between tumor and parenchyma compared to the TIC fitting analysis. This observation was confirmed by the following classification performance and the statistical analysis assessing the significance of the performance difference.

In the full 5-patient datasets, the case with pRCC subtype lacked contrast enhancement, hampering further TIC analysis. Table 2 shows the classification results for each individual ccRCC case and the results for the combined dataset. For all individual cases, as well as for the combined dataset, the spatiotemporal similarity analysis outperforms the TIC fitting analysis, achieving a maximum AUC of 0.96 on a single patient and all the AUC higher than 0.6 for the three similarity parameters on the combined dataset. In case 3, the poor classification performance can be attributed to the observation that elevated *ρ*, *r*, and *I* values, typically characteristic of tumor regions, were also detected in portions of the parenchymal area. The statistical analysis shows that spatiotemporal similarity analysis significantly outperforms in the scenario of RCC diagnosis with a *p-*value = 0.0103 (*p*-value < 0.05).

**Table 2:** Area under the receiver-operating-characteristic curve (AUC) for pixel classification of four ccRCC cases

Parameter	Case 1	Case 2	Case 3	Case 4	Total
$\:\varvec{\kappa\:}$	0.61	0.40	0.66	0.29	0.50
*AT*	0.89	0.80	0.65	0.35	0.68
*FWHM*	0.20	0.42	0.40	0.79	0.45
$\:\varvec{\rho\:}$	0.82	0.82	0.57	0.69	0.64
$\:\varvec{r}$	0.78	0.95	0.56	0.74	0.67
$\:\varvec{I}$	0.84	0.96	0.54	0.70	0.67

## Discussion

In this pilot study, we investigated the potential of CUDI for primary RCC diagnostics by implementing dedicated preprocessing steps including spatial resolution regularization, motion correction and SVD filtering, optimizing the CUDI algorithms and validating the results with pixel-based classification. The preliminary results demonstrate that the spatiotemporal similarity analysis outperforms the TIC fitting analysis in differentiating tumor and parenchyma regions, encouraging us to extend the dataset with a large number of reliable DCE-US acquisitions.

Compared to our previous experience on DCE-US acquisitions in the prostate, the DCE-US acquisitions in the kidney are more complex and heterogeneous, possibly because the image quality of the kidney can be affected by fat thickness, kidney size, tumor location and motion of surrounding organs. This increases the challenge for performing quantitative analysis. Limiting motion is beneficial for accurate CUDI analysis. The kidney was scanned transabdominally, which is more prone to motion artifacts as compared to the transrectal access employed for prostate imaging.

In clinical routine, clinicians prefer to use enhancement patterns of kidney DCE-US acquisitions to give a preliminary diagnosis, by describing the wash-in and wash-out of UCAs in the kidney [29]. These could be reflected by the parameters obtained from TIC fitting analysis, such as AT and FWHM, as well as time-to-peak and peak intensity as mentioned in [30]. However, the complexity and heterogeneity of kidney DCE-US acquisitions may hamper a robust and accurate analysis when only the temporal evolution of individual TICs is considered. This further emphasizes the importance of spatiotemporal analysis of neighboring TICs.

In our five acquisitions, the histological pRCC subtype can be easily differentiated from the ccRCC subtype due to a lack of enhancement in pRCC, hampering the feasibility of our CUDI analysis. The poor blood perfusion characteristics of the pRCC subtype are reflected into hypo-enhanced DCE-US imaging, as also reported in [16, 31]. For ccRCC, a heterogeneous hyperenhancement appears generally in the tumor regions, enabling the CUDI analysis. The obtained parametric maps demonstrate the difference between tumor and parenchyma regions, especially the higher spatiotemporal similarity values in the tumor regions, which is in line with our results on prostate cancer [19, 21, 22]. Tumor-driven angiogenesis is characterized by increased microvascular density and higher tortuosity [8, 32], which limits the dispersion of UCAs in the local measurement region. By modelling the UCA transport kinetics as a convective-dispersion process, lower dispersion is associated with higher similarity between neighbouring TICs [19, 21, 22]. Thus, spatiotemporal similarity analysis provides an indirect indicator of local dispersion, enabling tumor region detection. However, the (micro)vascular architecture in the kidney is complex, consisting of visible large vessels and dense microvessels. The TICs extracted from the large vessel regions show a high recirculation peak intensity and faster appearance time, which may influence the TIC fitting quality and the accuracy of extracted parameters such as AT and wash-in rate. The fitting quality directly influences the number of valid pixels for TIC analysis, which may further affect the classification performance. On the contrary, the spatiotemporal similarity analysis focuses on the shape similarity between neighboring TICs in a local region (kernel) without requiring TIC fitting, which can alleviate the impact of the presence of the high recirculation peak in individual TICs. Moreover, motion affects neighbouring TICs in a similar manner, especially in a local region; spatiotemporal similarity analysis is thus less affected by motion artefacts. This may explain why the spatiotemporal similarity analysis outperforms the TIC fitting analysis. In case 3, high similarity values also appear in the delineated parenchyma region, resulting in poor classification performance. It is hard to explain the reason based on the available ultrasound and CT images; therefore, histopathological results are necessary to shed some light into this peculiar case. Although the CUDI results obtained in this study can be interpreted by the underlying physiology of tumor-driven angiogenesis and the physics of the convective-dispersion process, the limited dataset constrains the generalizability of these findings in the context of RCC diagnosis. This limitation stems from three primary factors. First, the heterogeneity of RCC subtypes must be considered in CUDI analysis. Our dataset, being relatively small, only encompassed ccRCC and pRCC subtypes, leaving the applicability of CUDI to other subtypes unexplored. While ccRCC appears suitable for CUDI analysis, further validation of the diagnostic significance remains necessary based on histopathological reference and expanded ccRCC datasets, particularly given the unexplained poor classification performance observed in certain individual cases. Second, the UCA perfusion process in the kidney involves multiple phases, such as renal cortical enhancement and the final whole-kidney perfusion. This pilot study did not account for the potential impact of these multiphasic perfusion patterns on CUDI analysis, which may represent a significant limitation in diagnostic performance. Third, the complex microvascular architecture of the kidney, comprising both macroscopically visible vessels and dense microvascular networks, yields TICs with varying shapes. Future investigations with larger datasets should explore how optimization of the CUDI analysis for different vessel sizes could enhance both its accuracy and generalizability in the diagnosis of RCC.

In this pilot study, the patients were under anesthesia when scanning, which is challenging for clinical routine, especially for point-of-care ultrasound. Therefore, the potential of CUDI on routine kidney DCE-US acquisitions should also be investigated and proper measures should be taken to compensate for respiration motion. The recent development of 3D ultrasound imaging techniques can be beneficial for the mitigation of errors due to out-of-plane motion. Indeed, allowing for a more complete visualization of the kidney boundaries, 3D ultrasound imaging can lead to improved registration and compensation of motion artifacts due to respiration and free-hand scanning. Moreover, 3D imaging can provide comprehensive information on the hemodynamics of the whole kidney, describing more accurately the intrinsic behavior of blood flow and UCA perfusion in the kidney; therefore, we can directly model the 3D behavior of UCA transport through the kidney as a convective-dispersion process, which may allow us to extract more imaging markers, such as velocity vectors, dispersion, and vector-derived UCA transport tractography [33, 34]. In addition to hemodynamic parameters, ultrasound localization microscopy based on 2D and 3D DCE-US has recently been proposed to achieve resolutions beyond the diffraction limit in microvascular imaging, by detecting sparsely-distributed UCA microbubbles, tracking the centroids of their point spread functions over subsequent frames to reconstruct the microvascular networks where the microbubbles flow through. Several metrics related to the structure of the microvasculature can be extracted from ultrasound localization microscopy, such as vessel diameter, vessel density, vessel tortuosity quantified by distance metric, and fractal dimensions revealing the network complexity [35–38]. These may assess the tumor-associated angiogenesis in the kidney. However, the limited temporal resolution of most ultrasound scanners used in clinical routine as well as the motion artifacts during the scanning still hampers the implementation of ultrasound localization microscopy with regular clinical acquisitions in patients. Based on a set of quantitative parameters, our previous work also confirms that multiparametric ultrasound imaging achieved by training a machine-learning model to combine complementary parameters outperforms individual CUDI parameters for prostate cancer diagnosis whether using 2D or 3D imaging [39–41]. Hence, it is worth investigating in future studies the performance of multiparametric ultrasound imaging of the kidney, especially using histopathological results as the ground truth for tumor detection and subtype classification. While CE-CT is currently recommended for RCC diagnosis, the aforementioned advancements in ultrasound imaging and analysis techniques, combined with the inherent advantages of ultrasound imaging, including portability, high spatial resolution, real-time imaging capability, radiation-free operation in both static and dynamic imaging, along with its cost-effectiveness, establish a solid foundation for the clinical translation of CUDI as a point-of-care diagnostic tool for kidney cancer. Specifically, the portability of ultrasound systems, coupled with their cost-effectiveness, significantly reduces barriers to point-of-care diagnosis, making it accessible in diverse clinical settings. The high spatial resolution and real-time imaging capabilities enable visualization and hemodynamic analysis of tumor vascularity and perfusion patterns, providing both structural and functional information that complement tissue characterization. Furthermore, the radiation-free nature of ultrasound eliminates concerns about radiation exposure during dynamic acquisitions, a limitation inherent to CE-CT. In general, these advance the field of ultrasound-based cancer diagnostics.

## Conclusion

Our preliminary results show the potential of CUDI for solid RCC diagnostics, encouraging us to extend the dataset with a large number of reliable DCE-US acquisitions and corresponding histopathological results for further validation. Moreover, the recently developed 3D ultrasound imaging together with multiparametric image analysis techniques are worth investigating in the scenario of point-of-care diagnosis of kidney cancer.

## Data Availability

The research data are confidential due to privacy restrictions.

## Abbreviations

RCC: Renal cell carcinoma
DCE-US: Dynamic contrast-enhanced ultrasound
UCAs: Ultrasound contrast agents
CUDI: Contrast-ultrasound dispersion imaging
ccRCC: Clear-cell RCC
pRCC: Papillary RCC
chRCC: Chromophobe RCC
CT: Computed tomography
CE-CT: Contrast-enhanced computed tomography
TICs: Time-intensity curves
SVD: Singular value decomposition
κ: Dispersion-related parameter
AT: Appearance time
FWHM: Full-width at half-maximum
ρ: Spectral coherence
*r*: Temporal correlation
*I*: Mutual information
AUC: Area under the receiver-operating-characteristic curve
T: Tumor
P: Parenchyma
